# Case report: value of gene expression profiling in the diagnosis of atypical neuroblastoma

**DOI:** 10.1186/s13104-017-2724-4

**Published:** 2017-08-17

**Authors:** Anne C. Harttrampf, Qingrong Chen, Eva Jüttner, Julia Geiger, Gordon Vansant, Javed Khan, Udo Kontny

**Affiliations:** 1 Division of Pediatric Hematology and Oncology, Department of Pediatrics and Adolescent Medicine, Faculty of Medicine, Medical Center-University of Freiburg, Mathildenstr. 1, Freiburg, Germany; 2 Center for Biomedical Informatics and Information Technology, National Cancer Institute, NIH, Bethesda, MD 20892 USA; 3 Kiel Pediatric Tumor Registry, Department of Pediatric Pathology, University of Schleswig Holstein, Kiel, Germany; 4 Imaging Department, University Children’s Hospital Zurich, Zurich, Switzerland; 5 EPIC Sciences, San Diego, CA 92121 USA; 6 Oncogenomics Section, Genetics Branch, Center for Cancer Research, National Cancer Institute, NIH, Bethesda, MD 20892 USA; 7 Division of Pediatric Hematology, Oncology and Stem Cell Transplantation, Department of Pediatrics and Adolescent Medicine, University Medical Center, Aachen, Germany

**Keywords:** Neuroblastoma, Nephroblastoma, Gene expression profiling, Case report

## Abstract

**Background:**

Nephroblastoma and neuroblastoma belong to the most common abdominal malignancies in childhood. Similarities in the initial presentation may provide difficulties in distinguishing between these two entities, especially if unusual variations to prevalent patterns of disease manifestation occur. Because of the risk of tumor rupture, European protocols do not require biopsy for diagnosis, which leads to misdiagnosis in some cases.

**Case presentation:**

We report on a 4½-year-old girl with a renal tumor displaying radiological and laboratory characteristics supporting the diagnosis of nephroblastoma. Imaging studies showed tumor extension into the inferior vena cava and bilateral lung metastases while urine catecholamines and MIBG-scintigraphy were negative. Preoperative chemotherapy with vincristine, actinomycine D and adriamycin according to the SIOP2001/GPOH protocol for the treatment of nephroblastoma was initiated and followed by surgical tumor resection. Histopathology revealed an undifferentiated tumor with expression of neuronal markers, suggestive of neuroblastoma. MYCN amplification could not be detected. DNA-microarray analysis was performed using Affymetrix genechip human genome U133 plus 2.0 and artificial neural network analysis. Results were confirmed by multiplex RT-PCR.

**Results:**

Principal component analysis using 84 genes showed that the patient sample was clearly clustering with neuroblastoma tumors. This was confirmed by hierarchical clustering of the multiplex RT-PCR data. The patient underwent treatment for high-risk neuroblastoma comprising chemotherapy including cisplatin, etoposide, vindesine, dacarbacine, ifosfamide, vincristine, adriamycine and autologous stem cell transplantation followed by maintenance therapy with 13-cis retinoic acid (GPOH NB2004 High Risk Trial Protocol) and is in complete long-term remission.

**Conclusion:**

The use of gene expression profiling in an individual patient strongly contributed to clarification in a diagnostic dilemma which finally led to a change of diagnosis from nephroblastoma to neuroblastoma. This case underlines the importance of gene-expression profiling in the correct diagnosis of childhood neoplasms with atypical presentation to ensure that adequate treatment regimens can be applied.

**Electronic supplementary material:**

The online version of this article (doi:10.1186/s13104-017-2724-4) contains supplementary material, which is available to authorized users.

## Background

Neuroblastoma and nephroblastoma belong to the most common malignant solid tumors in childhood. In both cases children might present with a distended abdomen. Although there is a considerable overlap of symptoms in the initial presentation such as abdominal distension, loss of appetite, nausea or hypertension, children with nephroblastoma usually are in a better clinical condition and are slightly older with a peak incidence of 2–3 years [1]. A palpable, painless abdominal mass may be the only symptom present. In contrast, as neuroblastoma is metastasized in almost 50% at initial diagnosis, these children experience symptoms such as fever and anemia related to pancytopenia due to bone marrow involvement as well as weight loss and pain more frequently. Nevertheless, in some cases it may be difficult to distinguish neuroblastoma from nephroblastoma with standard diagnostic procedures but this is crucial with regard to optimal therapeutical management of these tumors [2, 3]. We describe a 4½ year old girl with neuroblastoma misdiagnosed as nephroblastoma based on the constellation of radiological and laboratory findings. Gene expression profiling and multiplex RT-PCR strongly supported the final diagnosis of neuroblastoma, underlining the importance of including molecular techniques in the diagnosis of childhood neoplasms with atypical presentation.

## Case presentation

**Fig. 1:**
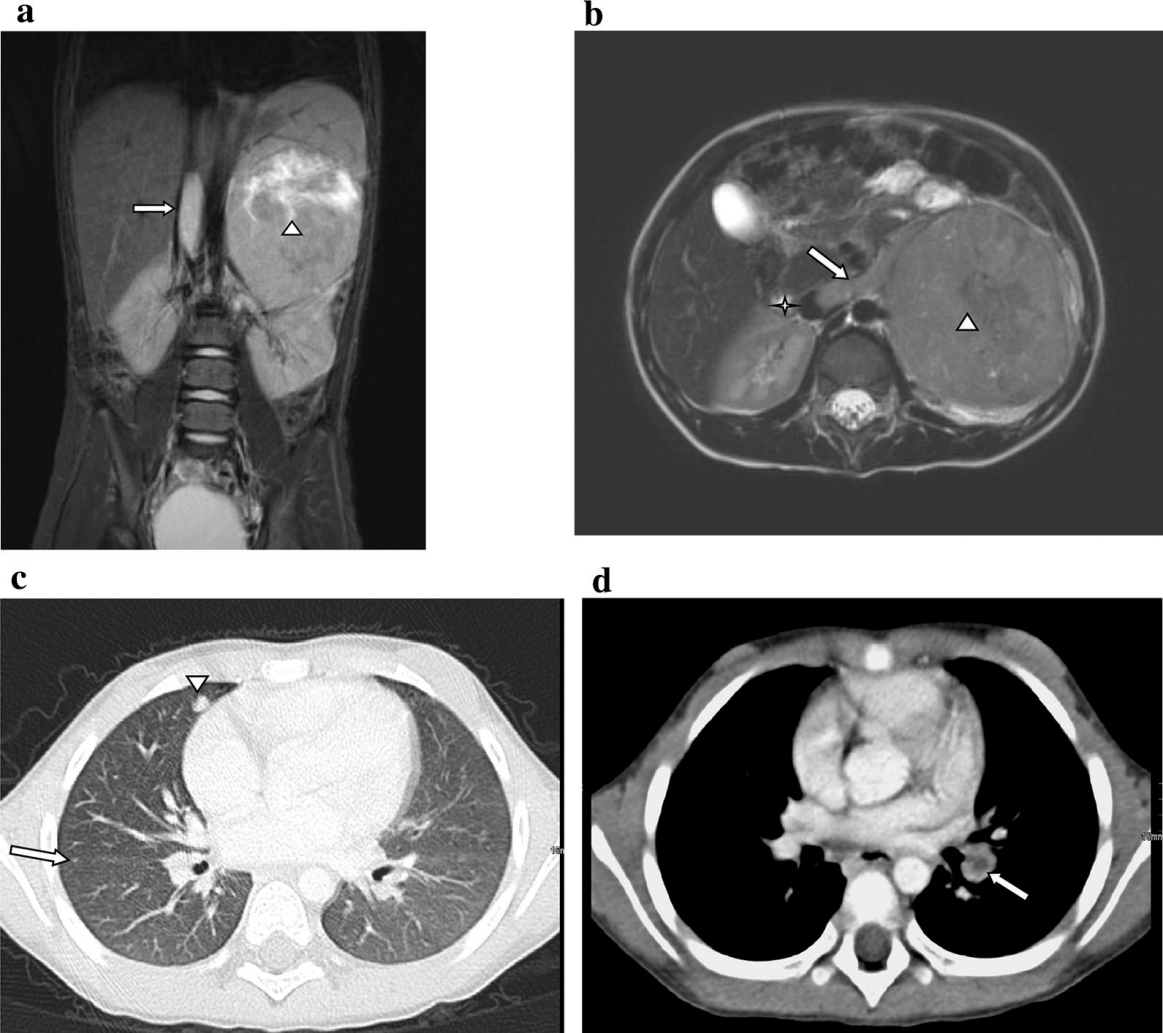
MRI abdomen at initial diagnosis (T2-weighted images). **a** (Coronal). Left-sided abdominal mass (*triangle*) arising from the upper pole of the left kidney and tumor thrombus in the vena cava inferior (*arrow*). **b** (Transversal). Main tumor formation (*triangle*) and tumor cone in the left vena renalis (*arrow*) extending to the vena cava inferior (*asterisk*). Computed tomography scan of the lungs. **c** The *arrows* indicate the presence of lung metastases in the middle lobe (*reverse triangle*) and right inferior lobe close to the pulmonary fissure (*arrow*). **d** Embolic occlusion (*arrow*) of the left inferior lobe artery by either a thrombus or tumor

**Fig. 2:**
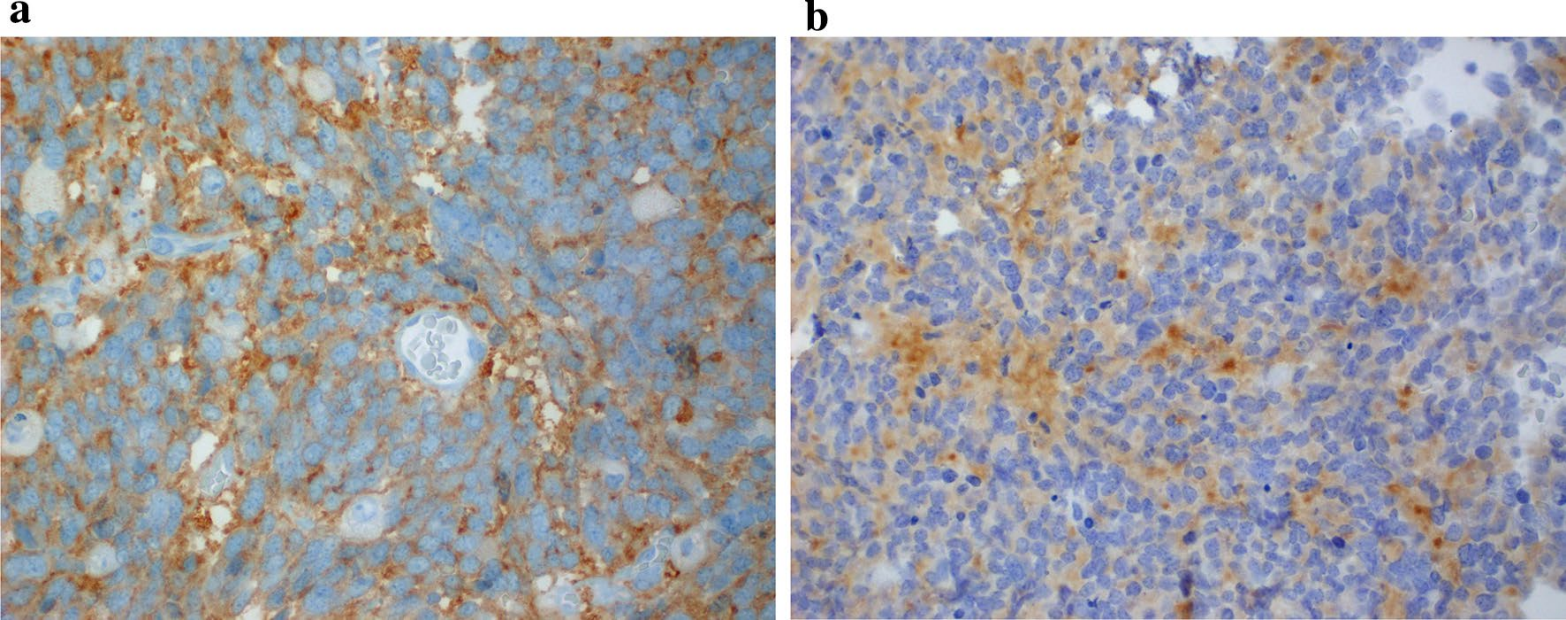
Immunohistochemistry of the primary tumor. **a** Expression of synaptophysin (×400). **b** Expression of VMAT (×400)

**Fig. 3:**
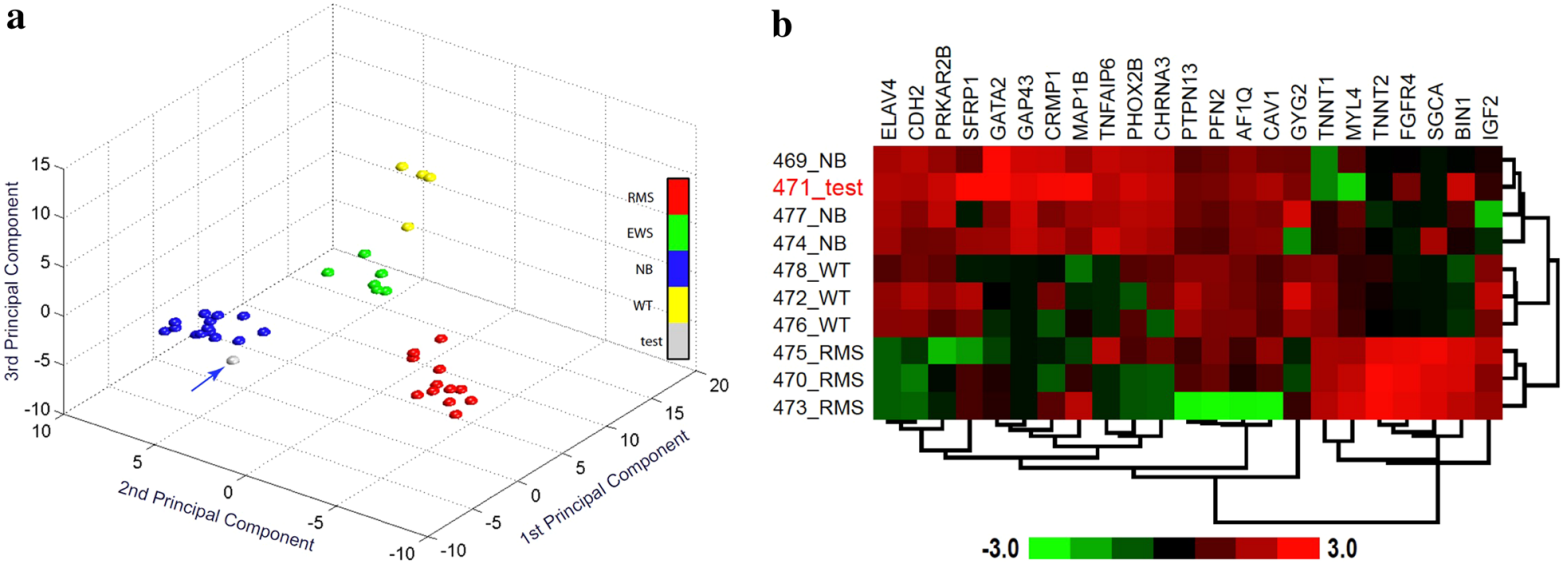
Gene expression profiling of the test patient tumor. **a** Loading plot of top three principal components of the 37 tumor samples (1 test patient sample, 12 RMS, 6 EWS, 14 NB and 4 WT) using 84 genes shows that the patient sample (test as *grey*) is closely clustering with NB. Affymetrix genechip human genome U133 plus 2.0 genechip is used to generate the data. RMS is depicted as *red circles*, EWS as *green*, NB as *blue* and WT as *yellow*. **b** Hierarchical clustering of 10 samples (1 test patient sample, 3 RMSs, 3 NBs and 3 WTs) and a subset of 23 genes out of a panel of 39 genes known to be differentially expressed in SRBCT using multiplex RT-PCR assay data shows that the test sample is clustering with NB. *Each row* represents a sample, and *each column* a gene. A pseudocolored representation of z-scored log_2_ ratio is shown

A 4½-year old girl presented with an abdominal mass and a 6-week-history of weight loss, decreased appetite and subsequent development of fever and abdominal pain. Physical examination and vital signs revealed no further pathological findings except of pallor, a reduced nutritional status and a slightly elevated temperature. Abnormal laboratory parameters indicative of a tumorous or inflammatory process are presented in Table 1. Urine catecholamines (homovanillic acid, vanillylmandelic acid) were not elevated. Abdominal magnetic resonance imaging (MRI) demonstrated an 8.8 × 7.9 × 9.4 cm retroperitoneal mass located at the superior pole of the left kidney with the tumor capsule passing into the renal capsule despite a non-infiltrative growth-pattern as shown in Fig. 1a (coronal image; marked by triangle). No vascular encasement or calcification was seen, but the tumor extended into the left renal vein (Fig. 1b, transversal image; marked by arrow) and the inferior vena cava (Fig. 1a, arrow; Fig 1b, asterisk). Except of enlargement of ipsilateral lymph nodes up to 9 mm no other tumor manifestations within the abdomen were seen. Chest X-ray and echocardiogram were normal, but chest computed tomography (CT) identified several bilateral pulmonary lesions strongly suggestive of metastases, the largest measuring 6 × 4 mm in the medial segment of the middle lobe (Fig. 1c; marked by reverse triangle and arrow). Further lesions were seen in the posterior segment of the right superior lobe and in the dorsobasal segment of the left inferior lobe. Both inferior lobe arteries showed evidence for an occlusion by either a thrombus or particles of the intracaval tumor cone (Fig. 1d; marked by arrow). Meta-iodobenzylguanidine scan was negative as were bone scan and cranial MRI. Based on the imaging and laboratory findings the tumor was suspected to be a nephroblastoma and a biopsy omitted due to the presumed risk of tumor rupture. Preoperative chemotherapy over 6 weeks according to the SIOP2001/GPOH-protocol with weekly intravenous administration of vincristine (1.5 mg/m^2^), actinomycine D (45 µg/m^2^) and adriamycine (50 mg/m^2^) was initiated, accompanied by continuous intravenous anticoagulation with unfractionated heparin (100 IU/kg/24 h) with respect to intravasal tumor manifestation. As abdominal MRI after 2 weeks of treatment displayed a constant renal tumor size but an expansion of the IVC thrombus the anticoagulation regime was changed to subcutaneous low-molecular-weight heparin twice daily (1 mg/kg/24 h, then escalated to 1.5 mg/kg/24 h aiming at factor anti-Xa-levels of 0.4–0.8 μmol/l). After 4 weeks of chemotherapy, MRI scans showed a slight regression of both the primary tumor mass and the IVC thrombus. After completion of 6 weeks of preoperative chemotherapy, the pulmonary metastases and the emboli in the inferior lobe arteries had regressed on chest CT. Radical tumor-nephrectomy, extraction of the caval thrombus and regional staging-lymphadenectomy were performed without major complications. Histopathology revealed clustering small tumor cells, partially arranged in pseudorosettes with focal calcifications, invading the left kidney. Immunohistochemistry was positive for synaptophysin, a specific marker of neuronal and neuroendocrine tumors and VMAT2, expressed in the CNS and sympathetic postganglionic neurons [Fig. 2a: expression of synaptophysin (400×); Fig. 2b: expression of VMAT (400×)]. PAN-cytokeratin and WT1, two major markers for nephroblastoma were negative as was desmin, a mesenchymal marker which can be positive in blastema-predominant nephroblastoma. These results further supported the diagnosis of undifferentiated neuroblastoma. The MYCN gene which is the most relevant genetic biomarker in neuroblastoma predicting poor prognosis was not amplified in this specimen. In order to substantiate the diagnosis gene-expression profiling and multiplex RT-PCR were done. Comparison of the gene expression profile using Affymetrix genechip human genome U133 plus 2.0 and artificial neural network (ANN) analysis of the patient’s sample with 36 tumor samples (12 rhabdomyosarcoma/RMS, 6 Ewing’s sarcoma/EWS, 14 neuroblastoma/NB and 4 Wilms’-Tumor/WT specimen) revealed that the patient sample (test as grey) was closely clustering with neuroblastoma (Fig. 3a; Additional file 1: Table S1). Hierarchical clustering of 10 samples (1 patient test sample, 3 RMSs, 3 NBs and 3 WTs) using a subset of 23 genes out of a previously established panel of 39 genes to distinguish small round blue cell tumors confirmed by multiplex RT-PCR showed that the patients’ sample was clustering with neuroblastoma tumors [4]. A pseudocolored representation of z-scored log_2_ ratio is shown (Fig. 3b). At that time the NSE had decreased to 20.2 μg/l (normal <17 μg/l), urine and serum catecholamines were still undetectable. Bone marrow aspiration and biopsy did not show any malignant cells. The following antineoplastic treatment comprised 6 cycles with alternating i.v. administration of either cisplatin/etoposide/vindesine or dacarbacine/ifosfamide/vincristine/adriamycin according to the GPOH NB2004 High Risk Trial Protocol. Intensification by high-dose, myeloablative chemotherapy with melphalan/carboplatin/etoposide and autologous stem cell rescue and a maintenance therapy with 13-cis retinoic acid p.o. over 10 months followed. Neither laboratory nor imaging investigations have been indicative of a tumor recurrence since and the child is well more than 4 years after completing treatment. Timeline summarizing patient management according to CARE Guidelines is provided in Additional file 2.

**Table 1:** Abnormal laboratory values at initial admission of the patient

Test	Result	Reference range	Units
LDH	670	104–311	U/l
Ferritin	146	12–60	ng/ml
NSE	260.2	<17	µg/l
C-reactive proteine	65	≤5	mg/l
D-dimers	422	≤130	µg/l
Hemoglobin	10.4	11.0–15.0	g/dl
MCV	71	75–87	fl

## Conclusion

Neuroblastoma and nephroblastoma both belong to the family of embryonic tumors and are the most important differential diagnoses to consider in malignant abdominal childhood tumors. European treatment protocols favour the administration of neoadjuvant chemotherapy in the management of nephroblastoma due to the substantial risk of tumor rupture during biopsy. A histology-proven diagnosis will generally not be available until several weeks after the start of antineoplastic treatment. The pre-histological differentiation of retroperitoneal tumors by radiological assessment based on certain diagnostic criteria (e.g. calcifications, vessel encasement, cysts) therefore plays a crucial role with regard to the management of further diagnostic procedures, operative treatment and chemotherapy. However, a marginal risk for misdiagnosing these patients on the basis of radiological imaging remains, especially in tumors that are located at the upper pole of the kidneys.

A retrospective analysis of 1609 patients registered in German neuroblastoma trials showed that 1.8% had been treated preoperatively as nephroblastomas [3]. Importantly, these patients have an inferior 5-year-EFS compared to patients treated according to neuroblastoma protocols from diagnosis on (0.40 vs. 0.64). These results especially held true for the subgroup of stage III patients (5-year EFS 0.27 vs. 0.68) whereas in the stage IV group the outcome was similarly dismal (5-year EFS 0.25 vs. 0.31). The inferior outcome was attributed to the observed unfavourable biology of these tumors rather than to the fact that appropriate neuroblastoma treatment was not primarily given.

In our case clear imaging findings indicative of neuroblastoma were absent but tumor expansion to the inferior vena cava was seen. So far, only few patients have been described in the English literature, reflecting the sparsity of this form of neuroblastoma extension [5–7]. In contrast, 4–10% of nephroblastoma patients show evidence for an intracaval thrombus [8]. Treatment recommendations include preoperative chemotherapy to induce regression of the tumor thrombus as reported in the majority of patients [9, 10].

Next to locoregional lymph nodes the organ most frequently involved by metastatic Wilms’ tumor is the lung. Our patient presented with bilateral pulmonary metastases, putting further weight on the diagnosis of a nephroblastoma. In contrast, although more than 50% of neuroblastoma patients have widespread disease at diagnosis, lung metastases are rather uncommon. In a large cohort of patients with stage IV neuroblastoma from the International Neuroblastoma Risk group database (n = 2808) the incidence of lung metastases at diagnosis was 3.6%. Patients with lung manifestation rather had prognostically relevant LDH elevation and MYCN-amplification compared to the group of stage IV patients without pulmonary metastases (82.1% vs. 67% and 53.9% vs. 29.1%, respectively) [11].

In more than 90% of neuroblastoma patients either homovanillic acid or vanillylmandelic acid are elevated [12] and MIBG-scan is positive in 90–95% of patients [11]. Neuroblastoma patients preoperatively misdiagnosed as nephroblastoma are more likely to present with normal catecholamine metabolites (39% vs. 80%) and less likely to have a positive MIBG scintigraphy (71% vs. 89%) in contrast to the group of neuroblastoma patients that were initially correctly diagnosed and treated [3]. Contrasting our case, an inverse correlation of catecholamine levels and amplification of the MYCN protooncogene has been described, showing that MYCN-positive tumors have rather low urinary VMA and HVA despite a comparably larger tumor volume [13, 14]. Interestingly, a mini case series by Gaetan et al. describes three neuroblastoma patients with intracaval tumor thrombus two of which had a similar constellation of both MYCN negativity and absence of elevated urine catecholamines [7].

Histopathology revealed clustering small tumor cells, partially arranged in pseudorosettes with focal calcification present. No definite conjunction with the renal tissue could be seen. Immunohistochemistry in our case stained clearly positive for synaptophysin (Fig. 2a) and vesicular monoamine transporter type 2 (VMAT2) whereas markers typically found in the majority of nephroblastomas were absent (Fig. 2b) [15, 16]. These findings were consistent with the diagnosis of undifferentiated neuroblastoma, which represents the rarest of three categories that neuroblastomas are classified into according to the Shimada classification. In a large series of 3501 newly enrolled patients in the COG Neuroblastoma studies undifferentiated neuroblastoma accounted for 4.5% and frequently associated with poor prognosis biomarkers such as advanced disease or MYCN amplification [17, 18]. However, Wilms’ tumors containing focal neural elements have been sporadically described in the literature with ganglion cells being the most prevalent neural element [19, 20]. Hussong et al. report on a familial series of four nephroblastomas all displaying histologic and immunohistochemical characteristics of focal neural differentiation, i.e. rosette formation and ganglion cells as wells as positive immunoreactivity for chromogranin, synaptophysin, NSE and vimentin. We therefore could not formally rule out the possibility of a nephroblastoma with heterologous cellular differentiation in our patient. MYCN was not amplified to serve as an important criterion of distinction between neuro- and nephroblastoma. From the clinical point of view, after having completed the 6-week neoadjuvant chemotherapy, slight tumor regression was observed in the primary tumor as well as in the metastatic sites. On the one hand this might have been due to tumor heterogeneity containing responding and resistant cellular compartments but also to the fact that two of the three drugs given (vincristine and adriamycine) are also part of neuroblastoma treatment.

To accurately discriminate between tumors of similar histological appearance but different origin, biology and clinical behaviour is an ongoing research topic. In the field of SRBCT and the overlap of nephroblastoma and neuroblastoma two recent reports propose immunohistochemical markers, cyclin D1 and PHOX2B, to be reliable for distinction of neuroblastoma from other malignancies [21, 22]. Different autoantibody signatures obtained from peripheral blood were detected with high sensitivity and specificity in untreated nephroblastoma and neuroblastoma patients, making this approach particularly interesting in the absence of histological samples [23]. In the case described in this study previous establishment of diagnostic signatures to distinguish SRBCT by gene expression analysis and a multiplex RT-PCR assay, the latter being extended for Wilms’ specific genes, allowed analysis of the patients’ tumor [24]. All results clearly showed that the patient sample clustered with neuroblastoma, further undermining the correct diagnostic reclassification and therapeutic restratification which led to a long-term complete remission in a high-risk neuroblastoma patient.

In summary, we present the clinical case of a patient with a renal tumor in whom conventional diagnostic procedures could not reliably differentiate between neuroblastoma and nephroblastoma. We conclude that genetic molecular approaches can be a valuable and precise supplemental tool for making the right diagnosis and instituting correct therapeutic management.

## Additional files

**Additional file 1: Table S1.** ANN diagnosis prediction.

**Additional file 2: Figure S1.** Timeline according to CARE Guidelines for case reports.

## Abbreviations

ANN: artificial neural network
CNS: central nervous system
COG: Children’s Oncology Group
CT: computer tomography
EWS: Ewing’s sarcoma
GPOH: German/Austrian/Swiss Society for Pediatric Oncology and Hematology
Hb: hemoglobin
HVA: homovanillic acid
i.v.: intravenous
IVC: inferior vena cava
LDH: lactate-dehydrogenase
MCV: middle cell volume
MIBG: meta-iodobenzylguanidine
MRI: magnetic resonance imaging
NB: neuroblastoma
NSE: neuronic specific enolase
p.o.: per os
RT-PCR: reverse transcriptase polymerase chain reaction
SIOP: International Society of Pediatric Oncology
SRBCT: small, round blue cell tumors
RMS: rhabdomyosarcoma
VMA: vanillyl mandelic acid
VMAT: vesicular monoamine transporter
WT: Wilms’ tumor
