# Supplementary material for: Case report: value of gene expression profiling in the diagnosis of atypical neuroblastoma
Source: BMC Res Notes. 2017 Aug 17;10:413. doi: 10.1186/s13104-017-2724-4 (PMC5561630; doi:10.1186/s13104-017-2724-4)
Supplement: Supplementary file 1 — Additional file 1: Table S1. ANN diagnosis prediction. [file 13104_2017_2724_MOESM1_ESM.doc]

| Supplemental Table 1. ANN diagnosis prediction | | | | | | |
| --- | --- | --- | --- | --- | --- | --- |
|  |  |  | ANN commitee vote | | | |
| Samples | Category | ANN Prediction | RMS | EWS | NB | WT |
| ARMS1 | RMS | RMS | 0.97 | 0.04 | 0.03 | 0.03 |
| ARMS2 | RMS | RMS | 0.97 | 0.04 | 0.03 | 0.04 |
| ARMS3 | RMS | RMS | 0.96 | 0.03 | 0.03 | 0.07 |
| ARMS4 | RMS | RMS | 0.97 | 0.03 | 0.03 | 0.03 |
| ARMS5 | RMS | RMS | 0.96 | 0.04 | 0.03 | 0.03 |
| ARMS6 | RMS | RMS | 0.96 | 0.03 | 0.05 | 0.04 |
| ARMS7 | RMS | RMS | 0.97 | 0.03 | 0.03 | 0.04 |
| ARMS8 | RMS | RMS | 0.91 | 0.14 | 0.03 | 0.04 |
| ERMS1 | RMS | RMS | 0.75 | 0.42 | 0.02 | 0.13 |
| ERMS2 | RMS | RMS | 0.97 | 0.03 | 0.04 | 0.04 |
| ERMS3 | RMS | RMS | 0.96 | 0.04 | 0.03 | 0.06 |
| ERMS4 | RMS | RMS | 0.95 | 0.07 | 0.04 | 0.05 |
| EWS1 | EWS | EWS | 0.09 | 0.93 | 0.03 | 0.07 |
| EWS2 | EWS | EWS | 0.06 | 0.95 | 0.05 | 0.05 |
| EWS3 | EWS | EWS | 0.06 | 0.95 | 0.06 | 0.05 |
| EWS4 | EWS | EWS | 0.09 | 0.94 | 0.04 | 0.11 |
| EWS5 | EWS | EWS | 0.04 | 0.93 | 0.11 | 0.05 |
| EWS6 | EWS | EWS | 0.05 | 0.95 | 0.05 | 0.05 |
| NB1 | NB | NB | 0.03 | 0.03 | 0.97 | 0.03 |
| NB2 | NB | NB | 0.03 | 0.03 | 0.97 | 0.03 |
| NB3 | NB | NB | 0.03 | 0.05 | 0.96 | 0.03 |
| NB4 | NB | NB | 0.03 | 0.05 | 0.96 | 0.03 |
| NB5 | NB | NB | 0.04 | 0.03 | 0.97 | 0.03 |
| NB6 | NB | NB | 0.03 | 0.03 | 0.97 | 0.03 |
| NB7 | NB | NB | 0.03 | 0.04 | 0.96 | 0.05 |
| NB8 | NB | NB | 0.05 | 0.04 | 0.97 | 0.04 |
| NB9 | NB | NB | 0.03 | 0.03 | 0.97 | 0.03 |
| NB10 | NB | NB | 0.02 | 0.06 | 0.95 | 0.06 |
| NB11 | NB | NB | 0.26 | 0.03 | 0.82 | 0.15 |
| NB12 | NB | NB | 0.03 | 0.04 | 0.97 | 0.04 |
| NB13 | NB | NB | 0.03 | 0.04 | 0.97 | 0.04 |
| NB14 | NB | NB | 0.03 | 0.04 | 0.97 | 0.03 |
| WT1 | WT | WT | 0.09 | 0.09 | 0.12 | 0.93 |
| WT2 | WT | WT | 0.11 | 0.12 | 0.08 | 0.93 |
| WT3 | WT | WT | 0.10 | 0.15 | 0.06 | 0.92 |
| WT4 | WT | WT | 0.17 | 0.17 | 0.09 | 0.91 |
| test | test | NB | 0.07 | 0.03 | 0.96 | 0.02 |
