# Supplementary material for: Case report: value of gene expression profiling in the diagnosis of atypical neuroblastoma
Source: BMC Res Notes. 2017 Aug 17;10:413. doi: 10.1186/s13104-017-2724-4 (PMC5561630; doi:10.1186/s13104-017-2724-4)
Supplement: Supplementary file 2 — Additional file 2: Figure S1. Timeline according to CARE Guidelines for case reports. [file 13104_2017_2724_MOESM2_ESM.pptx]

## Slide 1
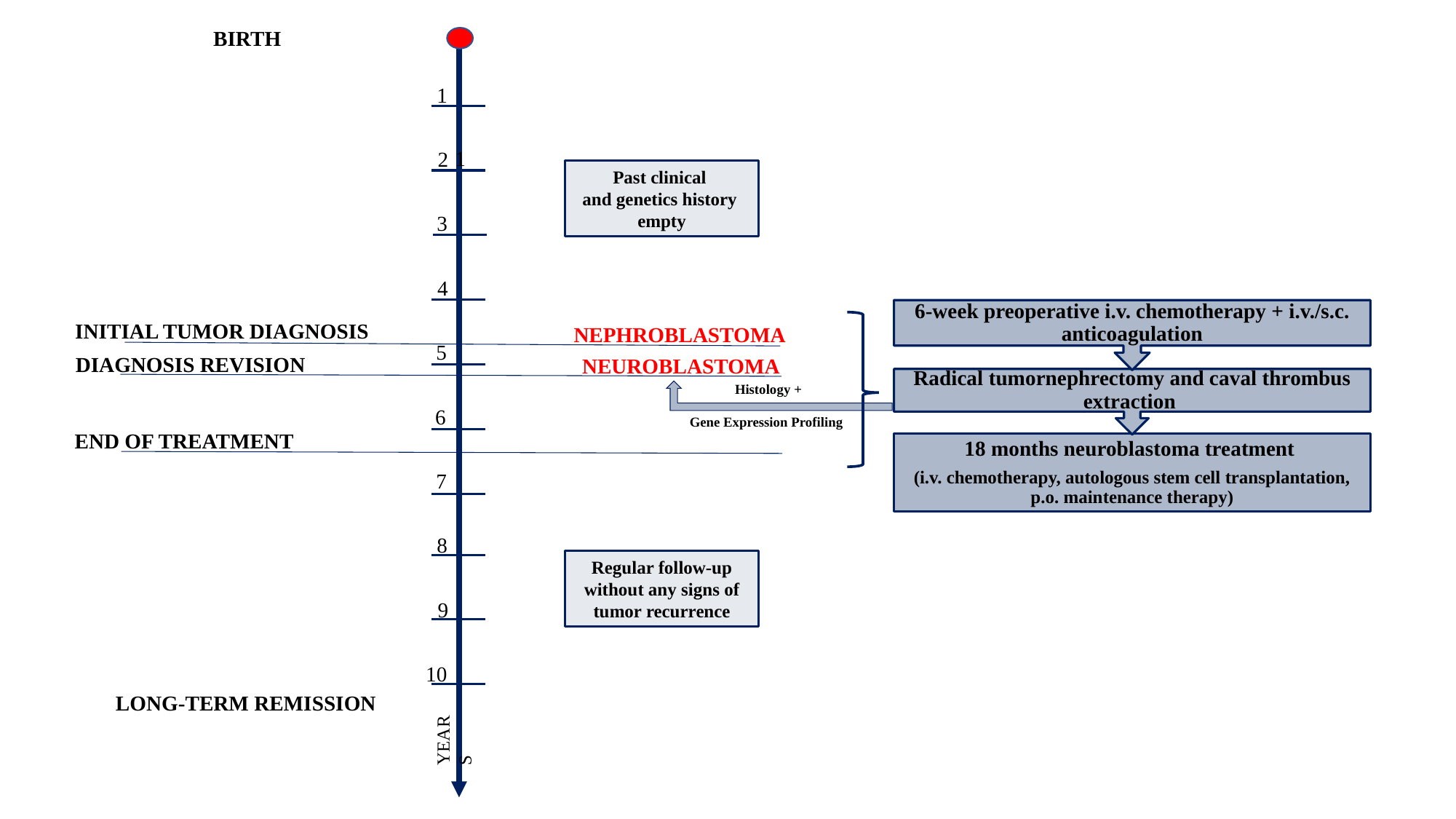

BIRTH
1
2
3
4
5
6
7
8
9
10
1
Past clinical
and genetics history
empty
INITIAL TUMOR DIAGNOSIS
NEPHROBLASTOMA
 DIAGNOSIS REVISION
 NEUROBLASTOMA
Histology +
Gene Expression Profiling
END OF TREATMENT
Regular follow-up without any signs of tumor recurrence
LONG-TERM REMISSION
YEARS
